# Quality monitoring in telestroke networks: real-world insights from survey response data and literature review

**DOI:** 10.3389/fstro.2026.1879025

**Published:** 2026-08-21

**Authors:** Ayush Agarwal, Christine Tunkl, Alexandra Krauss, Emily R. Ramage, Mirjam R. Heldner, Stefan T. Gerner, Teresa Ullberg, Leonardo A. Carbonera, Jatinder S. Minhas, Aristeidis Katsanos, Matias J. Alet, Abdul H. K. Y. Khan, Faddi Saleh Valez, Tamer Roushdy, Linxin Li, Bogdan Ciopleias, Zhe Kang Law, Radhika Lotlikar, Susanna M. Zuurbier, Maria G. Mosconi, Shirsho Shreyan, Shakti Shrestha, Gisele S. Silva, Annemarei Ranta

**Affiliations:** 1 Department of Neurology, All India Institute of Medical Sciences, New Delhi, India; 2 Department of Neurology, University Hospital Heidelberg, Heidelberg, Germany; 3 Florey Institute of Neuroscience and Mental Health, Heidelberg, VIC, Australia; 4 Department of Neurology, Inselspital, University Hospital and University of Bern, Bern, Switzerland; 5 Department of Neurology, University Hospital Erlangen, Erlangen, Germany; 6 Department of Neurology, Skane University Hospital, Lund University, Lund, Sweden; 7 Department of Neurology and Neurosurgery, MOVE Academic Research Organization, Hospital Moinhos de Vento, Porto Alegre, Brazil; 8 NIHR Leicester Biomedical Research Centre, Division of Cardiovascular Sciences, University of Leicester, Leicester, United Kingdom; 9 Department of Medicine (Neurology), McMaster University and Population Health Research Institute, Hamilton, ON, Canada; 10 Department of Neurology, Comprehensive Stroke Center, Fleni, Buenos Aires, Argentina; 11 Department of Neurology, Faculty of Medicine and Health Sciences, Universiti Putra Malaysia, Serdang, Malaysia; 12 Department of Neurology, The University of Oklahoma Health Sciences Center, Oklahoma City, OK, United States; 13 Neurology department, Faculty of Medicine, Ain Shams University, Cairo, Egypt; 14 Wolfson Centre for Prevention of Stroke and Dementia, Nuffield Department of Clinical Neurosciences, University of Oxford, Oxford, United Kingdom; 15 Department of Neurology, Faculty of Medicine, Transilvania University, Brașov, Romania; 16 Neurology Unit, Department of Medicine, Faculty of Medicine, Hospital Canselor Tuanku Muhriz, National University of Malaysia, Kuala Lumpur, Malaysia; 17 Department of Neurology, Deenanath Mangeshkar Hospital and Research Center, Pune, Maharashtra, India; 18 Department of Neurology, Antwerp University Hospital, Edegem, Belgium; 19 Internal Vascular and Emergency Medicine—Stroke Unit, University of Perugia, Perugia, Italy; 20 Rajshahi Medical College, Rajshahi, Bangladesh; 21 School of Pharmacy and Pharmaceutical Sciences, The University of Queensland, Brisbane, QLD, Australia; 22 Departamento de Neurologia da Universidade Federal de São Paulo (UNIFESP), São Paulo, Brazil; 23 Department of Medicine, University of Otago, Dunedin, New Zealand; 24 Department of Neurology, Wellington Hospital, Wellington, New Zealand

**Keywords:** quality monitoring, quality monitoring and evaluation, telestroke, telestroke medicine, telestroke models

## Abstract

**Background and aims:**

The objective of telestroke services is to enhance the quality of stroke care delivered to remote locations. The American Heart Association/ American Stroke Association (AHA/ASA) and the European Stroke Organization (ESO) have issued recommendations for quality monitoring in telestroke. To provide real-world insights, our study analyzed data on quality monitoring practices among telestroke networks.

**Methodology:**

This is a secondary analysis of a global telestroke survey previously conducted by our group. The focus of the current analysis was on reported quality metrics. We also reviewed articles from our prior systematic review (SR) of the literature on telestroke networks to extract quality metrics from published data and compared these with those reported in survey responses.

**Results:**

Eighty-eight of 254 networks responded to the survey. The SR analyzed 92 studies describing 64 acute telestroke networks across 17 countries. Quality monitoring was performed in 64 of 88 surveyed networks (72%), and it was described in all networks identified in the published literature. The most frequently reported performance indicator across both data sources was thrombolysis rate (93% in survey, 87% in SR). Other recommended indicators were reported less frequently in both the survey and the SR, with recanalization rate reported as 54% vs. 35%, discharge NIHSS as 65% vs. 1%, and in-hospital mortality as 78% vs. 31%, respectively.

**Conclusion:**

Our study is the first real-world insight into the state of quality monitoring in telestroke, both from peer-reviewed literature and self-reported data. The observed heterogeneity and low uptake of recommended quality indicators highlight the potential need for consensus on a standardized minimal quality monitoring dataset to facilitate feasible quality monitoring and comparison of outcomes across networks.

## Introduction

“Telestroke” was coined by Levine and Gorman in their seminal paper on the use of telemedicine for stroke care (Levine and Gorman, 1999). Telestroke is one of the most successful applications of telemedicine, as it helps provide stroke care from skilled stroke physicians to peripheral sites with limited or no stroke expertise (Wechsler et al., 2017). It has bridged geographical and temporal barriers to access to stroke care services, ensuring that stroke patients receive appropriate and timely stroke care from appropriate stroke physicians within the appropriate time frame (Wechsler et al., 2017). The primary aim of telestroke services is to improve the quality of stroke care provided to patients at remote sites. Therefore, quality monitoring to facilitate improvement within these networks is imperative to ensure that this aim is being achieved and that any gaps are addressed and monitored for ongoing improvement (Hubert et al., 2019). Stroke service quality monitoring involves the objective measurement of quality metrics (Wechsler et al., 2017). The American Heart Association/ American Stroke Association (AHA/ ASA) guidelines recommend that telestroke networks evaluate time to treatment, patient transfer data, outcome measures (patient outcome, stroke diagnosis and mimics, thrombolysis use, and safety measures [mortality and symptomatic intracranial hemorrhage]), patient and provider satisfaction, and telestroke technology quality (Wechsler et al., 2017). The ESO guidelines recommend the establishment of standard operating procedures (SOPs) [pre-hospital care, hyperacute stroke management, and stroke management in a stroke unit], professional training of staff members involved in telestroke, and data quality analysis (documentation of all generally recommended parameters in stroke care, specific telestroke parameters, and time delays in the telestroke workflow) (Hubert et al., 2019). Bladin et al. also conducted a comprehensive review and provided a minimum dataset of variables to evaluate telestroke programs systematically (Cadilhac et al., 2021).

Donabedian described that the quality of health care should be assessed along three interconnected constructs: structure, process, and outcomes (Donabedian, 1988). Structure denotes the settings where care occurs, process deals with the parameters involved in providing care, and outcome denotes the effects of care (Donabedian, 1988). The same construct applies to telestroke services. Previous studies have shown that the systematic collection of general stroke quality metrics and their analysis improves the quality of stroke care and has been widely implemented for this reason (Schwamm et al., 2009).

Our present study aims to provide real-world insights into which parameters are routinely collected in telestroke networks to guide quality improvement in telestroke care.

## Methodology

### Data collection

SURVEY: A previous study by our group on global telestroke coverage for acute stroke identified 254 telestroke networks through a comprehensive multi-level approach comprising a survey through scientific organizations and expert networks (Tunkl et al., 2025). A detailed structured survey was thereafter circulated to network leads, and the main survey findings have been published elsewhere (Tunkl et al., 2025). The survey contained questions about whether quality monitoring was performed within their respective networks and, if so, which parameters were measured from a given selection.

SYSTEMATIC REVIEW: Our group also conducted a systematic review of acute telestroke networks published in the past decade (Heldner et al., 2025). We reviewed these same studies for reported quality metrics and then compared these findings with those identified through our own real-world survey.

### Quality monitoring parameters

We divided quality monitoring parameters into three components:

- Structure-related
- Process-related
- Outcome-related

Structure-related parameters describe the settings where telestroke care occurs. These include standard operating procedures (SOPs), the mode of teleconsultation (real-time video examination/voice only/text only), documentation of teleconsultation, and the methods of image transfer for teleconsultation.

Process-related parameters describe the indices involved in providing care, such as the common stroke severity measure, the National Institute of Health Stroke Scale (NIHSS) score at admission, door-to-CT time, door-to-needle time, door-to-teleconsultation time, door-in-door-out time, thrombolysis rates, endovascular thrombectomy rates, duration of teleconsultation, and whether patients were transferred to a more specialized stroke center.

Outcome-related parameters describe the effects of care, i.e., NIHSS score 24 h after admission, in-hospital and 3-month mortality rates, and patient functional outcome using the modified Rankin Scale (mRS) score at 3 months, rates of stroke mimics, safety measures such as occurrence of intracranial or systemic bleeding, patient satisfaction, and caregiver satisfaction.

We classified countries into high-, middle-, and low-income countries based on the World Bank Classification (World Bank Blogs, 2025).

Quantitative data were described using descriptive statistics (number and percentage), and free-text responses were analyzed using inductive thematic analysis (conducted by AA and CT).

## Results

The survey was completed by 88 of the 254 networks, with a response rate of 34%, with 71% of responses from networks in high-income countries (HICs). Quality monitoring was performed by 64 of the 88 networks (72%) that responded to our survey (Table 1). The majority of these networks operated in Europe and the United States of America (25/64 [39%] and 13/64 [20%], respectively). A majority of the telestroke networks (87%) operated according to a set of SOPs.

**Table 1:** Quality parameters reported by global telestroke networks.

Quality indicators [*n* (%)]	Survey *N* = 64	Systematic review *N* = 64
1. Structure		
Standard operating procedures	56 (87)	x
Teleconsultation mode		
Voice only	6 (9)	
Real-time video examination	56 (87)	61 (95)
Messaging alone	2 (3)	
Image transfer	61 (95)	
WhatsApp/ Viber/ etc.	6 (9)	1 (1)
Specialized App	16 (26)	14 (21)
PACS	45 (73)	17 (26)
No information	x	34 (53)
Documentation of teleconsultation	59 (92)	x
2. Process		
Door-to-treatment time	61 (95)	56 (87)
NIHSS at admission	x	12 (18)
Door-to-imaging time	x	12 (18)
Door-to-consultation time	3 (4)	15 (23)
Inter-hospital transfer rate	x	32 (50)
3. Outcomes		
NIHSS 24-h after admission	1 (1)	3 (4)
NIHSS at discharge	42 (65)	1 (1)
Thrombolysis rates monitoring	60 (93)	56 (87)
Recanalization rate	35 (54)	23 (35)
Symptomatic ICH	X	36 (56)
In-hospital mortality	50 (78)	20 (31)
Secondary prevention measures	22 (34)	x
mRS at 3 months or 6 months	32 (50)	23 (35)

ICH- intracerebral hemorrhage; IQR- interquartile range; mRS- modified Rankin Scale; NIHSS- National Institute of Health Stroke Scale; PACS- Picture Archiving and Communication System.

The Systematic Review (SR) included 92 studies describing 64 acute telestroke networks across 17 countries. A majority of the networks originated from high-income countries (HICs) (*n* = 59, 92%). In contrast, only four networks were based in low- and middle-income countries (LMICs), accounting for 6% (*n* = 4/64) of all identified networks. The identified studies in the systematic review were predominantly observational in nature, and the level of evidence was generally low. The other systematic review findings, excluding quality metrics, are reported elsewhere (Heldner et al., 2025).

Both our survey and SR comprised 64 networks that reported data on quality monitoring.

Regarding structural quality metrics, 56 networks (87%) reported standard operating procedures in the survey. This information was not specifically reported in any of the studies analyzed in the systematic review. Teleconsultation modalities in the survey networks consisted of real-time video consultation in 56 networks (87%), voice-only communication in 6 networks (9%), and messaging alone in 2 networks (3%). SR networks reported real-time video examination in 61 networks (95%), while other teleconsultation modes were not specified. In both datasets, the majority of networks relied on the Picture Archiving and Communication System (PACS) for image transfer (survey: 73%, *n* = 45/64; review: 26%, *n* = 17), followed by specialized applications (survey: 26%, *n* = 16/64; review: 21%, *n* = 14) and messaging-based platforms such as WhatsApp or Viber (survey: 9%, *n* = 6/64; review: 1%, *n* = 1). Formal report generation for telestroke consultations was reported by 59 networks in the survey, whereas corresponding data were not available in the studies identified in the SR.

Regarding process indicators, door-to-treatment time was the most frequently documented quality indicator by 61 survey and 56 SR (95 and 87%, respectively) networks. Thrombolysis rate was monitored by 93% of survey networks (*n* = 60/64) and 87% of review networks (*n* = 56). Other metrics were less consistent across the two data sources, including door-to-imaging time (survey: none reported this; SR: 18%, *n* = 12/64) and door-to-consultation time (survey: 4%, *n* = 3/64; SR: 23%, *n* = 15/64). Inter-hospital transfer rates were reported in 50% (*n* = 32/64) of SR papers but by none in the survey; however, we did not offer this option as part of pre-specified selection options.

Outcome indicators focused on the in-hospital and long-term results of telestroke implementation. Outcome NIHSS was reported at various time points, including after 24 h and at discharge. NIHSS after 24 h was reported by one network in the survey (1%) and three networks in the SR (4%), whereas NIHSS at discharge was documented by 42 survey networks (65%) and 1 SR network (1%). In-hospital mortality was documented by 50 networks in the survey (78%) and by 20 networks in the SR (31%). Secondary prevention measures were not mentioned in any of the studies used in our SR but they were reported as quality metrics in 22 survey networks (34%). Functional outcomes assessed using the mRS at 3 or 6 months were reported by 32 survey networks (50%) and 23 SR networks (35%).

Comparing the frequency of quality monitoring in high-income countries’ (HICs) vs. low-middle-income countries’ (LMICs) networks, the survey revealed similar findings, with some metrics reported by 77% of responding networks in HICs vs. 63% in LMICs. All four published networks from the SR from LMICs reported IVT rate, followed by the mortality rate and complications (in three networks), and only one reported time metrics (Chutinet et al., 2019).

However, this lack of reporting in the literature does not necessarily indicate a lack of monitoring in practice.

## Discussion

Our survey found that 24 of the 88 telestroke networks (27%) did not perform quality monitoring, with similar rates in high- and middle-income countries. This could partly be due to recently established operational networks, most of which were based in LMICs, which may not yet have developed formal SOPs (Tunkl et al., 2025). Important telestroke clinical outcome parameters include NIHSS at 24 h/discharge, mortality, and 3-month functional outcome on the mRS (Zaidi et al., 2011; Saposnik et al., 2005). Both the AHA/ ASA and ESO recommend monitoring these outcome metrics (Wechsler et al., 2017; Hubert et al., 2019). However, our study found that these quality metrics were monitored by only 65, 78, and 50% of networks, respectively, which could be due to the transfer of the telestroke-treated patients to other centers and/or underappreciation of the importance of monitoring these outcome parameters to assess service quality.

Previous initiatives have successfully demonstrated that quality monitoring helps to improve care and processes: A study on telestroke from the Catalan region of Spain between 2013 and 2015 monitored baseline and 24-h NIHSS scores, thrombolysis rates, mortality, symptomatic ICH rate, and mRS at 3 months (López-Cancio et al., 2018). They demonstrated a progressive drop in telestroke door-to-needle time (DTN) between 2013 and 2015, an achievement the authors attribute to quality monitoring. A report from the Michigan Stroke Registry hospitals between 2022 and 2023 found that, among patients with acute ischemic stroke eligible for thrombolysis, telestroke evaluation had 61% higher odds of thrombolysis but 44% lower odds of DTN ≤ 60 min and prolonged door-in door-out times compared with those not evaluated by telestroke (Stamm et al., 2025). Identifying such issues has led to innovations in service improvement. For example, Kennedy et al. demonstrated that early activation of the teleconsultation pre-neuroimaging instead of post-neuroimaging, e-alert notifications, and storing the telestroke cart in the CT suite successfully addressed these issues (Kennedy and Stout, 2023; Hess et al., 2005). Similarly, Sambursky et al. (2026) recently showed that telestroke-associated emergency medical system prenotification, neurological assessment, and thrombolytic administration in the neuroimaging suite led to reductions in DNT and increased thrombolysis rates. Conversely, the use of German telestroke quality metric data found that earlier activation of teleconsultation results in increased volumes to the telestroke consultant for non-stroke cases, highlighting that our quality metric data can help individual services to optimize services to match local resources (Müller-Barna et al., 2014).

Determining whether a telestroke consultation requires transfer to another facility is an important outcome parameter. It helps to understand network practice patterns and the efficacy of care at the original hospital and to follow the functional outcome of the patient. A recent registry-based study analyzed interhospital stroke transfer rates after telestroke implementation and found that it was associated with an absolute decrease of 14.4% across all levels of stroke centers, offering important cost-savings to the health sector (Müller-Barna et al., 2014).

### System-level constraints and guidance on quality monitoring

Quality monitoring of key performance indicators in telestroke networks presents particular challenges for several reasons. First, responsibility for data monitoring is generally shared between the telestroke provider and recipient hospitals and may therefore be ambiguously defined. To facilitate this, shared data infrastructures that enable standardized data collection and analysis are required (i.e., telestroke provider monitoring the process components and recipient hospital tracking the outcomes) but are frequently lacking. Second, the patient pathway in telestroke systems often spans multiple hospitals, as patients are commonly transferred between facilities for further treatment. This fragmentation further complicates the systematic collection and attribution of outcome indicators. Third, in the absence of clear incentives or regulatory requirements, data monitoring is often deprioritized, as it is perceived as time-consuming and resource-intensive. Therefore, the absence of quality monitoring is not necessarily a matter of negligence but may instead reflect more systemic issues requiring solutions. Finally, despite mature telestroke implementation globally, quality monitoring remains disproportionately focused on time-to-treatment metrics, with limited attention to diagnostic accuracy, longitudinal outcomes, and network-level accountability. While capturing treatment delays is important for quality improvement, more effort is required to assess the other telestroke quality monitoring parameters. To address these challenges, it is recommended to establish formal agreements that clearly define responsibilities for quality monitoring. In addition, a standardized and feasible set of quality indicators should be agreed upon to enable consistent data collection across participating sites. A minimal dataset should include indicators on time metrics, safety, at least one clinical outcome parameter, and measures reflecting reliance on the telestroke network. In the absence of an internationally agreed minimal dataset, the global stroke community needs to establish a broadly accepted core set of indicators to support standardized quality monitoring across telestroke networks. We proposed the following minimum dataset for acute telestroke services, which could serve as a reference standard for future data collection and reporting (Table 2). More comprehensive recommendations on telestroke-specific quality monitoring are currently being developed by the WSO Telestroke Committee.

**Table 2:** Proposed minimum quality monitoring indicators for telestroke networks.

Structure	Teleconsultation mode Technical/ service failures
Process	Door-to-needle time (DNT) Intravenous thrombolysis rate Total number of stroke teleconsultations
Outcomes	In-hospital mortality Symptomatic ICH

Regular joint data review and feedback processes between provider and recipient hospitals are also essential. Such collaborative evaluation enables the identification of areas for improvement across the network and facilitates the joint development of targeted quality improvement measures.

### Strengths and limitations

Our study provides the first real-world insight into the state of quality monitoring in telestroke, both from peer-reviewed literature and from self-reported data, potentially capturing networks that have not published their data. However, self-reported data carry a risk of reporting bias and, more importantly, the relatively low response rate of 34% with over-representation in HIC (71%) likely introduced selection bias toward more structured and well-established networks, indicating that this may also have introduced bias toward desirability; therefore, quality monitoring may be even less common than what our study indicates. However, these biases are inherent to most current studies on healthcare quality evaluation, since they have been predominantly performed in high-income countries, an issue that should be urgently addressed. Finally, missing data in published studies may reflect reporting choices rather than the absence of monitoring. This indicates that standardized reporting for telestroke networks is highly needed.

## Future directions and conclusion

Our findings may help in the development of a standardized minimal dataset for telestroke networks that is suitable for a variety of resource settings to systematically track structural, process, and outcome metrics. It may also encourage comprehensive quality monitoring by motivating networks to implement broader quality monitoring, including clinical outcomes, treatment times, and patient-centered measures, to identify gaps and improve care. Finally, considerations may begin for the establishment of a telestroke network certification program at the national or global level, with quality monitoring as a key requirement, to promote high standards of care and enable benchmarking across institutions.

## Data Availability

The original contributions presented in the study are included in the article/Supplementary material, further inquiries can be directed to the corresponding author.
