# Supplementary material for: Quality monitoring in telestroke networks: real-world insights from survey response data and literature review
Source: Front Stroke. 2026 Aug 21;5:1879025. doi: 10.3389/fstro.2026.1879025 (PMC13541472; doi:10.3389/fstro.2026.1879025)
Supplement: Supplementary file 1 [file Supplementary_file_1.DOCX]

Telestroke Project - Questionnaire:

Explanatory note:

*We analyze all official and unofficial telestroke networks, which*

*- are used among healthcare professionals /* ***between hospitals*** *operating within the* ***acute inpatient hospital setting (<3 days after onset).***  *- are* ***based on any formal processes recognized by the hospitals.***

*We exclude: 
-  telerehabilitation networks and direct telemedicine consultations between doctors and patients
- private counseling / guidance / mentorship / advice on an irregular or friendship basis*

*- communication between resident and consultant within one hospital*

A - NETWORK CHARACTERISTICS

1. In which country is your hospital?

2. What is your role in the network?

a) Network coordinator

b) Provider of telestroke care

c) Caller / recipient of telestroke care

d) Other:

3. How many years have you been involved in this telestroke network?

a) <1 year

b) 1-5 years

c) >5 year

4. How many years has the network been in existence?

a._____

b. In process of setting up

5. What kind of hospital support is provided by your telestroke model? -*comments is to add description to each of the model – do you have the description of each of the module?*

a. Hub and spoke with connection
b. Hubless network
c. Other - please specify:

6. How many hospitals are connected in the network? - suggest adding number of hospitals

a. ____________-
b. not sure

7. What is the location of most connected sites? – need to provide option to tick > than 1 as may have combination

a. Rural
b. Urban
c. Semi-urban
d. Both
e. I don't know

8. Which of the following describes the setting of the originating hospital? – comment: what do we mean by originating hospital?

a. Academic, comprehensive stroke center
b. Non-academic, comprehensive stroke center
c. Secondary care center
d. Primary care center
e. No originating hospital exists
f. For-profit company provides consultation
g. Other - please specify:

9. Which of the following describes the setting of the remote / spoke hospital? – comment: may need to give description of remote hospital (and need to give option to choose more than 1)

a. Academic, comprehensive stroke center
b. Non-academic, comprehensive stroke center
c. Secondary care center
d. Primary care center
e. Other- please specify:

10. What is the purpose of tele-stroke? *Please tick all that apply.*

a. Prehospital care
b. Emergency consultation for (hyper-)acute stroke
c. Hyperacute management – thrombolysis
d. Hyperacute management – mechanical thrombectomy
e. Intracerebral hemorrhage - consultation
f. Stroke unit supervision
g. Rehabilitation
h. Other - please specify:

11. Which services are provided by your tele-stroke model? *Please tick all that apply.*

a. Support in thrombolysis and thrombectomy decision-making
b. Transfer for thrombolysis
c. Transfer for thrombectomy
d. Transfer for neurosurgical interventions
e. Transfer for stroke unit care or intensive care
f. Discussion of difficult cases
g. Other:

12. What are the operating times of telestroke care provision?

a. 24/7
b. Out of core - working hours (e.g. nights, weekends)
c. Special case advice only
d. Other:

13. How many telestroke consultation are provided on average by month (in the last year)? – to add numbers

a. < 10
b. 10 – 50
c. 50 – 100
d. > 100
e. sI don't know

C - WORKFLOW AND PROCESSES

14. What is the average time needed for one complete consultation?

a. <5 minutes

b. 5-20 minutes

c. 20-45 minutes

d. >45 minutes

15. Do you have a standardized protocol (SOP)?
a. Yes

b. No

c. I don't know

16. What are the components of a teleconsultation? *Please tick all that apply.*

a. Real-time video examination

b. Voice call (physician - physician)

c. Image transfer

d. Written documentation (referral letter)

e. Text messaging or whatsapp

17. Does your consultation include real-time video examination?

a. Yes

b. No

If so, what type of technology is used for the call?

a. Commercial phone apps like Whatsapp/Viber/etc
b. Specialized app for telemedical use
c. Specialized external camera devices
d. Other: ___________________

18. Does your consultation include image sharing (CT/MRI)?

a. Yes

b. No

If so, what type of technology is used for the image sharing?

a. Commercial phone apps like Whatsapp/Viber/etc
b. Specialized app for telemedical use
c. Regular hospital IT - system / PACS system
d. Specialized imaging transfer devices
e. Other: ___________________

19. Does your consultation include written documentation/reporting?

a. Yes

b. No

If so, what type of technology is used for documentation?

a. Commercial phone apps like Whatsapp/Viber/etc
b. Specialized app for telemedical use
c. Regular hospital IT - system
d. Other - please specify: ___________________

20. What follow-up is provided after telestroke care by the telestroke provider/the originating hub?

a. Follow up with the calling physician
b. Follow up with the patient
c. Only if required by the calling center
d. None
e. Other - please specify:

21. What were the (estimated) costs for setting up the system (App, devices)?

a. ____

b. I don't know

22. What are the annual maintenance costs

- for the technical devices / App __________________

- for human resources _________________________
- I don't know

23. What is the financial mode of the telestroke network?

a. Public health system resources

b. Private / Out-of-pocket payments by patients

c. Social insurance

d. Third-party support / sponsors

e. Mixed public/ private

f. Other - please specify:____________

B - HUMAN RESOURCES

24. How many doctors in your institution provide telestroke care?

a. __________
b. I don't know

25. Do these physicians fill full-time positions for telestroke care only or do they offer teleconsults in addition to their regular jobs?

a. Full-Time
b. Part-Time/ Add-on
c. I don't know
d. Other:

26. What is the qualification of the tele stroke consultant? *Please tick all that apply.*

i. Resident Neurology
j. Resident Internal Medicine
k. Consultant Neurology
l. Consultant Internal Medicine
m. Other:

27. What is the average number of years of clinical experience of the consultant?

a) <1 year

b) 1-5 years

c) >5 year

28. What is the qualification of the person seeking tele-consultation? *Please tick all that apply.*

a. Resident
b. Qualified physician (e.g. internal medicine)
c. Neurologist
d. Nurses
e. House officer
f. Paramedical staff
g. Emergency Physician
h. GP
i. Patients

D - PATIENTS AND PROCESS INDICATORS

*The following questions aim to assess some performance indicators, which are not available in all networks. It is no problem if you do not have these data, please give us an estimation then.*

29. Is the quality and outcome of the telestroke program monitored?

a. Yes
b. No
c. I don't know

If so, which performance indicators of consultations do you measure?

a. NIHSS at discharge

b. Recanalization rate

c. Door-to-treatment - Time

d. Door-in/Door-out-Time

e. In-hospital mortality

f. Measures of secondary prevention (e.g. ECG, antihypertensives...)
g. 3-months Follow - up (e.g. mRS)

h. Other - please specify:

s

30. Does your network conduct any research within your network based on telestroke?

a. Yes

b. No

c. I don't know

31. How many patients get thrombolysis via teleconsultation per month on average (in the last year)? a. _______________ (estimated / validated)

b. I don't know

32. How many patients are transferred to your center for EVT per month on average (in the last year)?

a. _______________ (estimated / validated)

b. I don't know

33. What is the average Door-to-Needle-Time via teleconsultation (in the last year)?

a. _______________ (estimated / validated)

b. I don't know

E - PERCEIVED STRENGTHS AND LIMITATIONS

34. Which barriers do you perceive in your telestroke network? *Please select all that apply.*

a. Legal issues
b. Issues with funding / financing
c. Technical issues (app/ devices) with communication
d. Technical issues with image transfer
e. Internet connection
f. Accountability in decision making
g. Time exposure
h. Too much documentation
i. No clear procedure/ protocol
j. Cooperation with caller
k. Other - please specify:

35. Which strengths do you perceive in your telestroke network?

a. Little time expenditure
b. Improved quality of care in remote hospitals
c. Less documentation
d. Shorten referral time
e. Able to get expert consultation
f. Other - please specify:

36. What is your overall level of satisfaction with the telenetwork model?

a. Very satisfied

b. Satisfied

c. Neutral

d. Dissatisfied

e. Very dissatisfied

37. Do you consider the quality of care provided by telecare in network is better, worse or equal to an onsite consultation?

a. Much better

b. Minimally better

c. Equal

d. Minimally worse

e. Much worse

F - PERSONAL DATA
*Please share your email-address and the name of the network/ city/ region on a voluntary basis. This will help us to verify information in case of multiple, divergent responses from a network. For all analysis, we will treat the information pseudonymized.*

Email-Address:

Name of city/ network:

Do you agree that your network is listed in the WSO registry and in a publication?

In case there are multiple networks in your country, please share their names and contact of their coordinator:

Do you agree to be contacted for future research questions?

- Yes

- No
